# Phantom Sensations Following Brachial Plexus Nerve Block: A Case Report

**DOI:** 10.3389/fneur.2018.00436

**Published:** 2018-06-08

**Authors:** Hannah G. Russell, Jack W. Tsao

**Affiliations:** ^1^Department of Neurology, University of Tennessee Health Science Center, Memphis, TN, United States; ^2^Children's Foundation Research Institute, Le Bonheur Children's Hospital, Memphis, TN, United States; ^3^Department of Neurology, Memphis Veterans Affairs Medical Center, Memphis, TN, United States

**Keywords:** frozen limb, phantom limb sensation, phantom limb pain, cortical remapping, brachial plexus injury, brachial plexus anesthesia, amputation, cortical reorganization

## Abstract

Following the administration of brachial plexus anesthesia for right thumb carpometacarpal arthroplasty with ligament reconstruction, a 54-year-old woman with all limbs intact developed phantom limb sensations, including the misperception of the placement of her right arm and frozen limb sensations in her fingers. Immobility of her fingers in a stacked position was experienced for ~3.5 days after surgery, and she described her phantom sensations as the hand experiencing “tingling” and feeling “heavy.” While the onset of these phantom sensations occurred almost immediately after administration of brachial plexus anesthesia, they lasted for ~69 h after anesthesia wear off, suggesting that cortical effects from denervation resolves much more slowly than initial remapping, giving insight into the mechanisms behind phantom limb sensations that are often experienced by amputees.

## Introduction

Following major limb amputation nearly all amputees will experience phantom limb sensations (PLS), and ~80% will experience phantom limb pain (PLP) (1). PLS have been described as non-painful feelings of a specific shape, movement, position, or temperature of the missing limb, and can include itching and tingling, while PLP is a term used to describe any severely uncomfortable feelings in the phantom limb (2).

Although the etiology behind PLS/PLP remains unknown, one of the leading theories is cortical remapping (3). The cortical remapping theory, otherwise known as the maladaptive plasticity theory, suggests that PLS and PLP arise from the invasion of cortical regions neighboring the zone within the primary sensorimotor cortex previously controlling the amputated limb (3). A direct correlation between the amount of phantom limb pain and the amount of cortical remapping has been found (4). Other theories are a dissociation between vision and proprioception (5) and proprioceptive memories (6). The visual-proprioception dissociation theory suggests that the disconnect between what the amputated limb looked like and how the phantom limb is perceived by the amputee currently is the cause of PLP, and a decrease in the disconnect between the visualization and proprioception of the phantom limb results in a decrease in PLP (5). The theory of proprioceptive memories suggests that memories of the limb's position prior to amputation remaining embedded in the subconscious after amputation contribute to PLP and frozen limb sensations (6).

While studies have been conducted to show the direct relationship between the amount of cortical reorganization and PLS and PLP (4, 7), they have not been able to concretely describe the changes that occur in the peripheral and central nervous systems after amputation. Brachial plexus avulsion injury (BPAI) is a result of the detachment of the nerves of the arm from the nerve roots of the spinal cord, resulting in partial or complete paralysis of the arm (8, 9). BPAI is very common after traffic accidents, and 30–80% of patients with BPAI develop tingling, electric shock, and burning neuropathic pain (10) similar to the PLP and PLS experienced in amputees (2). Over 80% of patients experience this chronic pain following complete BPAI (10, 11). One report described the case of a patient who experienced PLP and PLS after BPAI, even though the patient had an intact limb (12). In another recent case report, a BPAI patient experienced hand-to-face remapping and PLP in his intact, but denervated limb, suggesting that cortical reorganization had occurred following the injury and that the etiology behind the PLP and PLS after BPAI is similar to that experienced in amputees (13). Dorsal root entry zone lesioning has been used to effectively treat pain in both PLP patients and BPAI patients (14), suggesting that the formation of pain after BPAI is similar to that after amputation. Similarly, mirror therapy, which is commonly used to help treat PLP in amputee patients (15–17), has been reported to successfully treat the chronic pain experienced in BPAI patients (13, 18), giving further credence to the suggestion that PLP and PLS after amputation is very similar to the sensations and pain felt after BPAI (13).

The PLP and PLS experienced by both amputee and BPAI patients can be debilitating (19, 20), and a lack of understanding of the mechanisms behind PLP and PLS hinders the ability to develop successful treatments for patients (2, 20). Brachial plexus anesthesia (BPA) is a temporary deafferentation that is often administered for routine upper-extremity surgeries (21) and might serve as a model for the permanent deafferentation experienced in amputees. Therefore, studying patients undergoing BPA has the potential to aid in the understanding of the roles of the peripheral and central nervous systems and in PLP and PLS.

## Case report

**Figure 1:**
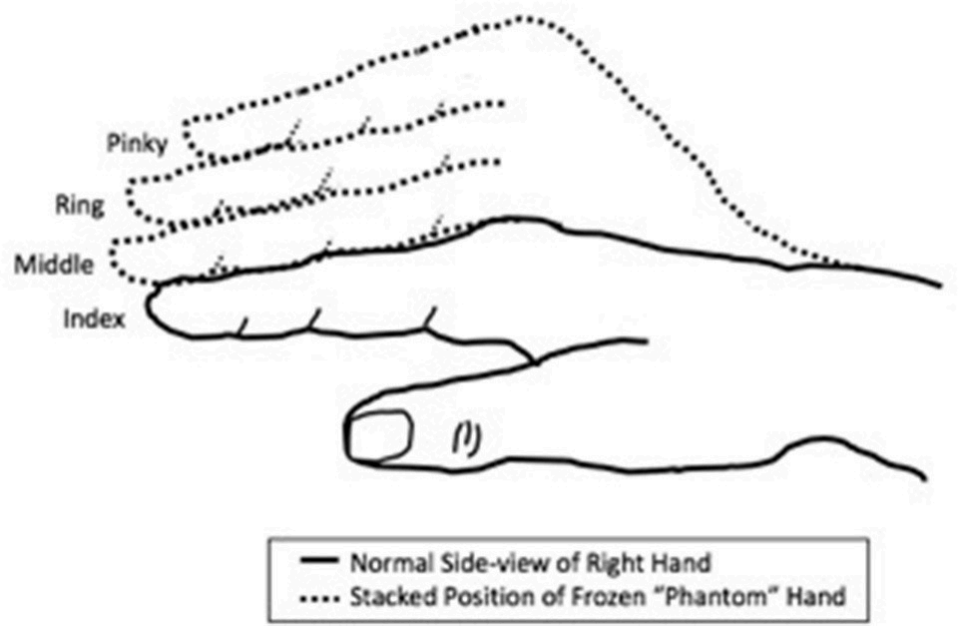
Schematic of frozen limb sensations experienced in the “phantom” hand of the patient. The fingers were experienced as being “stacked” on top of each other in a non-anatomic manner, rather than crossed over on each other.

A 54-year-old woman with all limbs intact received BPA in advance of right thumb carpometacarpal arthroplasty with ligament reconstruction. Immediately after BPA onset, she felt her right forearm and hand resting across her chest when it was hanging over the side of the gurney. After surgery, her right hand felt “heavy” with the fingers stacked vertically on top of each other, as shown in Figure 1. She began experiencing right thumb pain 14–16 h after the operation had been completed. However, the sensation of immobility of her 2nd through 5th digits in the stacked position lasted for ~3.5 days after surgery and 69 h after the anesthesia wore off. During this time, although the patient described the phantom sensations as being uncomfortable, she experienced no pain in the fingers. No nerve conduction studies were performed.

This study was carried out in accordance with the recommendations of the University of Tennessee Health Science Center. The procedure discussed in this report was not part of a research study but rather routine clinical care. The subject gave written informed consent for publication of her clinical details in accordance with the Declaration of Helsinki.

## Discussion

The etiology of phantom limb phenomena after amputation remains unknown. Phantom sensations and pain have also been described by individuals with intact, but denervated limbs, such as BPAI (12, 13). One study found that administering BPA to amputees with PLP quickly and significantly reduced both the amount of cortical reorganization and the amount of PLP experienced by the amputees, showing a direct relationship between the amount of PLP and cortical remapping (7). While the induction of BPA was found to improve PLP in some, others experienced no improvement in pain levels (7). Additionally, it has been reported that spinal anesthesia induced PLP in an amputee who did not previously experience phantom pain (22). Spinal anesthesia has also been reported to exacerbate the effects of PLP (23). The emergence of PLS under anesthesia in these studies, in addition to what we report here, demonstrates that, although anesthesia has variable effects on reducing PLP, it can rapidly induce phantom limb phenomena in both amputees and persons with intact limbs.

BPA, routinely administered for surgical procedures on the upper limb, is a temporary nerve blockade, and could be considered to be a model for the permanent deafferentation experienced by amputees. Although the patient discussed in this report has all limbs intact, the PLS, similar to those experienced in amputees, emerged within 10 min following onset of the anesthetic effect. The patient's feeling of her arm being in a position in a different area than the actual anatomic position has been reported previously (24). In a study examining phantom sensations after the administration of BPA, it was found that 94% of 77 patients with intact limbs who received an adequate amount of BPA for surgery on the upper limb experienced a feeling of a “phantom” arm resting on his or her chest or abdomen even though it was on the operating table (24).

What is unique about this case is the lingering of apparent frozen limb sensations even after wear-off of the anesthesia. The term “frozen limb” is used to describe the sensations of immobility of a phantom limb in a specific position (25). Although the etiology of frozen limbs is unknown, there have been multiple reports of amputees with phantom limbs “frozen” in the same position the limb was in prior to amputation (26–28). It has been postulated that frozen limbs occur due to proprioceptive memories that store the position of the previously-intact limb prior to amputation (6). However, while our patient experienced similar sensations to those experienced in amputees with frozen phantom limbs, the positioning of her immobile “phantom” fingers after BPA was not the same position of her fingers before the BPA, suggesting that the frozen limb sensations experienced by this patient were of a different proprioceptive memory and indicating that such de novo sensations can arise under BPA and likely represent a different cortical connection pathway than that activated by the last known anatomic position. In addition, although abnormal frozen phantom limb positions have been reported before (29), the stacking position of phantom limbs described by this patient is novel. Of note, although the position in which this patient's fingers were immobilized was abnormal and not anatomical, it was not painful.

While the mechanisms behind phantom limb pain and sensations are unknown, because the patient experienced the sensation that her denervated arm was in a new position soon after the administration of BPA, this suggests that the onset of PLS can occur extremely rapidly after denervation. Rapid remapping has been found to occur within minutes of deafferentation in humans (30), and it has been found that 72% of amputees experience PLP within 8 days after amputation (31). However, since the frozen sensations persisted roughly 69 h after the nerve blockade terminated, it is possible that the return of somatosensory reorganization back to its original state is a much slower process than the initial remapping. The extended persistence of phantom sensations after the wear-off of anesthesia is an important new finding because it suggests that phantom sensations can continue even after nerve functioning is recovered and that the remapping process back to its original state is slower than the initial remapping after denervation. This novel information could give insight into the cortical remapping theory and how it relates to phantom sensations experienced by amputees.

The findings from this patient suggest that PLS experienced by patients with intact limbs receiving routine BPA for upper-extremity surgeries could be studied as a model to help better understand the mechanisms and time course behind how phantom limbs, sensations, and pain arise in both amputee and BPAI patients. Further studies should be conducted to analyze the amount of cortical remapping and PLS that occurs in the setting of BPA. In addition, the positioning of the limb before and after denervation should be studied to better understand frozen limb sensations. This information would improve our understanding of how PLS and PLP arise.

## Author contributions

HR collected and analyzed the data and wrote the manuscript. JT supervised conduct of critical input into the drafting and editing of the manuscript and interpretation of the findings.

### Conflict of interest statement

The authors declare that the research was conducted in the absence of any commercial or financial relationships that could be construed as a potential conflict of interest.
